# Home versus hospital immunoglobulin treatment for autoimmune neuropathies: A cost minimization analysis

**DOI:** 10.1002/brb3.923

**Published:** 2018-01-26

**Authors:** Gwendal Le Masson, Guilhem Solé, Claude Desnuelle, Emilien Delmont, Marc Gauthier‐Darnis, Sophie Puget, Isabelle Durand‐Zaleski

**Affiliations:** ^1^ Neuromuscular Diseases Department University Hospital Pellegrin Bordeaux France; ^2^ Neuromuscular Diseases Department University Hospital L'Archet Nice France; ^3^ Neuromuscular Diseases Department University Hospital La Timone Marseille France; ^4^ Market Access and Pricing Department LFB Biomedicaments Les Ulis France; ^5^ International Scientific Affairs Unit LFB Biomedicaments Les Ulis France; ^6^ Public Health, AP‐HP University Paris Est Creteil, & ECEVE UMR 1123 Paris France

**Keywords:** cost, home care, immunoglobulins, immune neuropathies

## Abstract

**Background:**

Prior clinical trials have suggested that home‐based Ig treatment in multifocal motor neuropathy (MMN) and chronic inflammatory demyelinating polyradiculoneuropathy (CIDP) and its variant Lewis–Sumner syndrome (LSS) is safe and effective and is less costly than hospital‐administered intravenous immunoglobulin (IVIg).

**Methods:**

A French prospective, dual‐center, cost minimization analysis was carried out to evaluate IVIg administration (5% concentrated) at home versus in hospital with regard to costs, patients’ autonomy, and patients’ quality of life. The primary endpoint was the overall cost of treatment, and we adopted the perspective of the payer (French Social Health Insurance).

**Results:**

Twenty‐four patients aged 52.3 (12.2) years were analyzed: nine patients with MMN, eight with CIDP, and seven with LSS. IVIg (g/kg) dosage was 1.51 ± 0.43 in hospital and 1.52 ± 0.4 at home. Nine‐month total costs per patient extrapolated to 1 year of treatment were €48,189 ± 26,105 versus €91,798 ± 51,125 in the home and hospital groups, respectively (*p *<* *.0001). The most frequently reported factors for choosing home treatment were the good tolerance and absence of side effects of IVIg administration, as well as a good understanding of the advantages and drawbacks of home treatment (75% of respondents). The mRankin scores before and after switch to home treatment were 1.61 ± 0.72 and 1.36 ± 0.76, respectively (*p *=* *.027).

**Discussion:**

The switch from hospital‐based to home‐based IVIg treatment for patients with immune neuropathy represents potentially significant savings in the management of the disease.

## 1 INTRODUCTION

IVIg is considered as first‐line treatment of demyelinating peripheral neuropathies such as chronic inflammatory demyelinating polyneuropathy (CIDP), multifocal motor neuropathy (MMN), and their variants, such as Lewis–Sumner syndrome (LSS) (Joint Task Force of the EFNS and the PNS, 2010; Van den Bergh et al., 2010). Although all the mechanisms for IVIg efficacy in CIDP and MMN are not totally understood, at doses ranging from 1 to 2 g per kg per cycle, IVIg interferes with both the innate and adaptive immune systems (Anthony, Kobayashi, Wermeling, & Ravetch, 2011). Corticosteroids and/or plasma exchanges (Elovaara et al., 2008; Patwa, Chaudhry, Katzberg, Rae‐Grant, & So, 2012) are alternative first‐line treatment options. Most of these patients will require recurrent infusions of IVIg every 3 to 8 weeks, and 55% of CIDP patients are still treatment‐dependent after 18 months (Viala et al., 2010). Immunoglobulins rank among the top drug expenses for hospital in France. In addition to the high drug costs, IVIg treatments require the use of hospital resources for recurrent infusions. Home infusion has been used since the 1990s (Ochs et al., 1987; Ryan, Thomson, & Webster, 1988) and is now considered to be a safe alternative to hospital care. The possibility of providing safe home treatment with IVIg makes it possible to reduce treatment costs, make better use of hospital resources, and improve the patient's quality of life (QoL). The aim of this analysis was to estimate and compare the costs of home‐based *vs*. hospital‐based recurrent infusions.

## 2 PATIENTS AND METHODS

Patients currently treated for autoimmune neuropathy with IVIg in a hospital outpatient settings were recruited for a before–after study from two tertiary referral care centers in France.

### 2.1 Design

**Figure 1:**
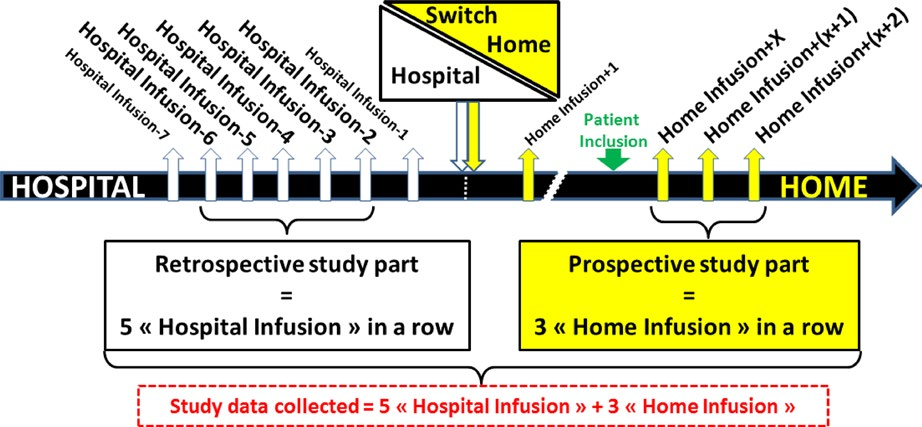
Study design

The analysis was designed as a before–after analysis, with each patient being his or her own control. The design was chosen for practical reasons, as the patients enrolled in this analysis were stable and their IVIg treatment costs did not vary over time prior to switching to home treatment (Figure 1). We compared the two IVIg treatment procedures in a cost minimization analysis, considering all direct costs to the healthcare system and community.

### 2.2 Patient selection

Patients from the two French referral centers were monitored before and after switch to home infusion. For patients to be eligible for home treatment, they had to:

1. Be 18 years or older.
2. Have one of the following conditions: chronic inflammatory demyelinating polyradiculoneuropathy (CIDP); multifocal motor neuropathy (MMN); and Lewis–Sumner syndrome (LSS).
3. Have received previous treatment with IVIg for a minimum of five cycles.
4. Be responders to IVIg and clinically stable.
5. Have received one home treatment for the same disease and agree to continue home treatment for at least three more cycles.

Patients were excluded if they were currently participating in another therapeutic trial, had combined neuropathy and monoclonal anti myelin‐associated glycoprotein (anti MAG) gammopathy, or were already alternating hospital and home treatments. After individual consent was obtained, patients were asked to rank their reasons for switching to home treatment out of the following: comfort, no previous history of adverse events, good tolerance of IVIg, autonomy, costs, and family organization.

### 2.3 Intervention

All patients had a caregiver (e.g., trusted family member) with them during the infusion, who had been trained on home management while the patient was in hospital. Before beginning home treatment, nurses in the referral facility were trained for IVIg infusion (Tegeline^®^, 5% freeze‐dried, sucrose‐stabilized) or had previous experience in administering and monitoring these treatments. Hospital pharmacy staff provided additional training in handling and transporting blood products. Vascular access was reviewed and clinical competency for the nursing staff achieved. An administration and surveillance protocol was established according to the recommendations of the manufacturer (LFB Biomedicaments). A routine blood workup (urea, creatinine, blood cell count, hemoglobin, ALT/AST, coagulation assessment, ESR, C‐reactive protein) was performed 24 hr prior to infusion and 24 hr after the end of the cycle as per current European guidelines (Elovaara et al., 2008). These tests are performed identically in all patients regardless of the place of treatment, and we did not report their costs in the comparison. Results were then systematically transmitted to the referral specialist for formal approval to proceed with the infusion. If premedication was used in the previous hospital infusion, the same protocol was used. The IVIg was administered using an infusion pump.

Prior to the home visit, the hospital pharmacy dispensed the IVIg directly to the nurse after the doctor orders and prescriptions were cross‐checked. The IVIg was packaged in a validated, temperature‐controlled cooler (monitored via a thermometer). Once at home, the patient was premedicated if this was part of the determined protocol. Venous access was achieved and baseline vital signs taken. The IVIg was administered following a predefined protocol (doses, infusion time, premedication). The patient status was monitored throughout the infusion. Side effects were reported to the principal investigator and to the pharmacy and recorded in the patient's medical chart. Once the infusion was complete, the IV line was discontinued and the patient was monitored for another 60 minutes to ensure that he or she was stable. Unused IVIg was returned to the pharmacy.

### 2.4 Data collection

Healthcare resource utilization both in the hospital and in the community was recorded. Data for the “before” phase (hospital‐based infusion) were extracted from the patient's medical charts, supplemented by the hospital's claims database. The claims database has linked records of all inpatient and outpatient admissions with a unique patient identifier. For all patients in the study, the following patient characteristics were recorded: age, sex, diagnosis, comorbidities, modified Rankin scale (mRankin) score, living conditions, profession, and eligibility for welfare benefits (Rankin, 1957).

Data for the “after” phase (home‐based infusion) were collected prospectively over a 9‐month period. Care pathway variables included hospital admissions (inpatient and outpatient), home visits from nurses or physicians, cost of the device used for home‐based IVIg treatment, costs of transporting Ig from the hospital to the patient's home, total number of cycles, and total doses of Ig, other medications, and transportation.

The primary endpoint was the cost of treatment. Costs were estimated from a payer perspective (French Social Health Insurance) over the hospital‐based and home‐based infusion periods and adjusted to a 12‐month period.

Patients with chronic conditions are eligible for 100% coverage for all medical expenditures related to their condition and do not have a copayment in France. Resource valuation used the national tariffs for all healthcare resources. Immunoglobulin was priced using the official national price per gram. Travel costs for consultations and hospital admissions are also covered and were assessed based on the actual means of transportation chosen: medical vehicle (tariff for ambulance) or personal vehicle (national distance‐adjusted compensation). Time costs and potential loss of productivity were not included in the cost calculations. Table 1 summarizes the unit costs (https://www.ameli.fr/infirmier/exercice-liberal/facturation-remuneration/tarifs-conventionnels/tarifs, last accessed on November 22, 2017; https://libermedical.fr/nomenclature-des-actes-infirmiere-liberale-cotation-soins-infirmiers.html, last accessed on November 22, 2017; https://www.ameli.fr/medecin/exercice-liberal/facturation-remuneration/tarifs-generalistes/tarifs-metropole, last accessed on November 22, 2017; http://www.atih.sante.fr/tarifs-mco-et-had, last accessed on November 22, 2017; https://www.legifrance.gouv.fr/affichTexte.do?cidTexte=JORFTEXT000022128154, last accessed on November 22, 2017; http://www.apidel.fr/IMG/pdf/NGAP-PERFUSION-v5.pdf, last accessed on November 22, 2017; https://www.ameli.fr/paris/assure/remboursements/rembourse/transport/transport, last accessed on November 22, 2017).

**Table 1:** Unit costs of healthcare and community resources in France

	Hospital IVIg	Home IVIg infusion	Source
Home nurse visit:	NA		Social health insurance schedule (https://www.ameli.fr/infirmier/exercice-liberal/facturation-remuneration/tarifs-conventionnels/tarifs, last accessed on November 22, 2017; https://libermedical.fr/nomenclature-des-actes-infirmiere-liberale-cotation-soins-infirmiers.html, last accessed on November 22, 2017)
Treatment charge		3.15 €	
Preparation		9.45 €	
Installation		12.60 €	
Infusion		44.10 €	
Monitoring		6.30 €+ 18 .90 €/hour	
Discontinuation		6.30 €	
Personal transport x2		2.50 €	
GP visit	NA	23 €	Social health insurance schedule (https://www.ameli.fr/medecin/exercice-liberal/facturation-remuneration/tarifs-generalistes/tarifs-metropole, last accessed on November 22, 2017)
Outpatient admission for IVIg infusion (excluding IVIg costs)	1,927 €	NA	DRG tariff (http://www.atih.sante.fr/tarifs-mco-et-had, last accessed on November 22, 2017)
Inpatient admission for IVIg infusion (excluding IVIg costs)	2,675 €	NA	DRG tariff, code 23M091 (http://www.atih.sante.fr/tarifs-mco-et-had, last accessed on November 22, 2017)
IVIg per gram	39 €/g	39 €/g	National price (https://www.legifrance.gouv.fr/affichTexte.do?cidTexte=JORFTEXT000022128154, last accessed on November 22, 2017)
Dispensation fee	NA	22 €	Social health insurance schedule (https://www.ameli.fr/infirmier/exercice-liberal/facturation-remuneration/tarifs-conventionnels/tarifs, last accessed on November 22, 2017; http://www.apidel.fr/IMG/pdf/NGAP-PERFUSION-v5.pdf, last accessed on November 22, 2017)
Infusion pump		225 €	Social health insurance schedule, code LPP1164778 (https://www.ameli.fr/infirmier/exercice-liberal/facturation-remuneration/tarifs-conventionnels/tarifs, last accessed on November 22, 2017)
Disposable infusion supplies per kit		75 €	Social health insurance schedule (https://www.ameli.fr/infirmier/exercice-liberal/facturation-remuneration/tarifs-conventionnels/tarifs, last accessed on November 22, 2017)
IVIg transportation	NA	9 €	Social health insurance schedule (https://www.ameli.fr/infirmier/exercice-liberal/facturation-remuneration/tarifs-conventionnels/tarifs, last accessed on November 22, 2017)
Patient transportation: patient's vehicle €/km medical transportation/taxi	0.32 € 13.28 € + 0.85 €/km	NA	Social health insurance schedule (https://www.ameli.fr/paris/assure/remboursements/rembourse/transport/transport, last accessed on November 22, 2017)

GP, general practitioner; IVIg, intravenous immunoglobulin.

The secondary endpoints assessed the quality of life of patients treated at home and the impact of home treatment on patients’ autonomy. The impact of home treatment on autonomy was assessed by comparing the mRankin score before and after the switch to home treatment.

No ethics approval was required for this analysis as only routine care was given. Patients gave consent to the data collection and did not oppose the use of data already recorded in administrative database. All patient information was anonymized in the database using coded identification numbers, and no information in the database could be backtraced to reveal the patient's identity.

The analysis was undertaken between 2012 and 2014. All resources were valued at 2016 prices, and costs are reported in €2016.

### 2.5 Statistical analysis

#### 2.5.1 Sample size calculation

We calculated that 22 patients would provide 80% power, with a two‐sided alpha level of 0.05, to detect a 50% relative decrease in the yearly total cost of IVIg treatment as compared to the in‐hospital treatment (estimated total hospital cost of €100,000). This calculation method was conservative: Cost distributions tend to be skewed and follow a gamma distribution. Sample size calculations based on differences in means were found to be very conservative, giving numbers which substantially exceed the required power (Cundill & Alexander, 2015).

Given the need for continued monitoring of patients’ disease, we did not expect any missing value on the use of healthcare resources.

The unit of analysis was the patient, using an intention‐to‐treat analysis based on period. All costs of the “before” period were attributed to hospital‐based treatment, and all costs of the “after” period, including hospital admissions, were attributed to home‐based infusion. The total cost of each period was divided by the number of months and multiplied by 12 to obtain a yearly patient cost. This calculation assumed that all patients were receiving a stable IVIg maintenance regimen. Outliers were not removed.

Continuous data were reported as mean ± *SD*, and the paired Student's *t* test was employed when comparisons were made for parametric data. Nonparametric data were analyzed with the paired Wilcoxon test, and we used 1,000 bootstrap replications to estimate the 95% confidence interval of the costs and cost difference. All tests were two‐tailed, and a *p* value of <.05 was predetermined to represent statistical significance. Analyses were carried out using the SAS version 9.1 (SAS Institute, Cary, NC).

## 3 RESULTS

The two centers identified 24 patients who were monitored for 9 months. Table 2 shows the baseline characteristics of the study patients. The mean age was 52.3 years (±12.2) with a male to female sex ratio of 2:1. Of the 24 patients included, 14 were working full‐ or part‐time, one patient was unemployed, and the others were retired. Six of 24 patients used implantable venous access devices, and the remaining 18 used IV lines. Patients were monitored for an average of 8.53 (±2.85) months and 4.96 (±2.86) months during the “before” and “after” periods, respectively.

**Table 2:** Patient characteristics at baseline. Values are indicated in mean (Standard deviation) unless otherwise specified

	*N*/mean/standard deviation
Age (years)	52.3 (12.2)
Sex ratio M/F	2/1
Underlying disease (*N* patients)	
CIDP	8
MMN	9
Lewis–Sumner syndrome	7
Disease duration prior to switch (years)	8.7 (4.6)
Ig treatment duration prior to switch (years)	8.1 (4.4)
Living conditions = family/alone (*N* patients)	21/3
Other treatments (*N* patients)	
Plasma exchange	2
Corticosteroids	9
Immunosuppressants	10
Inpatient/outpatient treatment prior to switch (*N*)	12/12
mRankin score at baseline	1.61 (0.72)
Number of cures during the follow‐up period (9 months)	7 (2.3)

CIDP, chronic inflammatory demyelinating polyradiculoneuropathy; MMN, multifocal motor neuropathy.

During the “before” period, in hospital, the doses ranged from 37.2 to 203.7 grams per treatment cycle (1–2 g/kg), with a mean dose of 114.81 (±32.87) grams dosed every three to four weeks as per the protocols in the centers. Before the switch, 12 patients were treated as inpatients and 12 as outpatients. Patients traveled on average 20 km to go to the hospital.

After the switch to home treatment, Ig was delivered directly to the patients in six cases, and to a nurse in the remaining 18. Infusion devices were IV line and electric pump, and infusion times at home averaged 3 hr. Doses ranged from 58.5 to 222.3 g per treatment cycle, with a mean dose of 119.38 (±38.14) g.

During the “after” period (at home), two patients were admitted to hospital and one patient discontinued home treatment for personal reasons.

**Figure 2:**
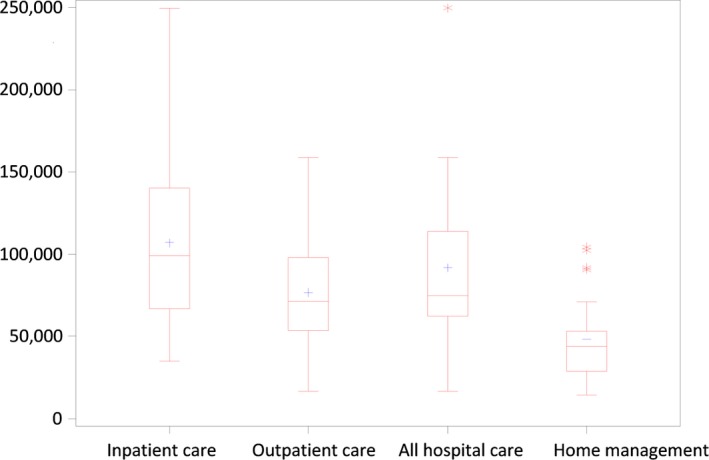
Annualized difference in total cost between hospital and home management, by type of patient and choice of hospital care (in‐ vs outpatient). All costs are in €

Overall costs per patient per cycle before the switch amounted to €11,473 (95% bootstrapped CI [€9,701;€13,175]) and were reduced to €5,712(95% bootstrapped CI [€4,879;€6,209]) after the switch. The reduction in cost per cure was €5,761 (95% bootstrapped CI [€4,019; €7,645]). Detailed costs are presented in Table 3 and Figure 2. The reduction in average total costs extrapolated to 1 year was €43,609 (95% CI [€25,800; €61,418]) for home treatment compared to hospital treatment. When costs were analyzed by disease type, higher treatment costs and cost difference were found in patients with LSS and CIDP compared to patients with MMN. Determinants for switching from hospital to home treatment were explored via a self‐administered questionnaire. The most frequently reported factors (75% of respondents) were the good tolerability and absence of side effects of in‐hospital IVIg administration, as well as a good understanding of advantages and drawbacks of home treatment. The mRankin scores before and after switch to home treatment were 1.61 (±0.72) and 1.36 (±0.76), respectively (*p *=* *.027). Only five of 24 patients found no, minor, or moderate improvement in quality of life after the switch to home treatment, and 19 found significant or major improvement. Reasons for satisfaction included fewer commutes and time spent in the hospital, greater comfort, gain in professional or leisure time, and presence of family.

**Table 3:** Resource utilization and treatment costs in € before and after the home treatment switch. Costs in € were estimated per cure and per year by extrapolating the yearly number of cures. *p* values were calculated using paired Student's *t* test and Wilcoxon paired test for nonparametric analyses. Values are means (standard deviation) and medians

	Hospital IVIg	Home IVIg	*p* value
Entire population *N* = 24			
GP visits (€)			
Number of visits	0.3 (0.2)	0.33 (0.2)	
Cost	6.8 (3.6)	7.7 (4.1)	
Hospital admissions			
Hospital days (50% inpatient and 50% outpatient for hospital IVIg patients)	2.8 (1.1)	0.08 (0.5)	<.0001
Hospital costs (€)	6,853 (3,442)	536 (219)	
Immunoglobulin			
Dosage (g/kg weight/cure)	1.51 (0.43)	1.52 (0.40)	.066
Costs (€)	4,500 (1,401)	4,975 (1,609)	
Costs of IVIg dispensation and infusion (€) (1 of each for each cure)	NA		
Nurse intervention		162.4 (111)	
Dispensation fee		22.0 (0.0)	
Costs of infusion pump (€) (1 pump for the entire period)	NA	225 (63.4)	
Costs of disposable infusion supplies (€) – (1 kit per cure at home)	NA	75 (162)	
Costs of IVIg transportation (€) (1 transport per cure at home)	NA	8.9 (37.7)	
Patient transportation			
Average in km	36 (27)		
Average cost (€)	113 (116)		
Total cost per patient per cure (€)	11,473 (4,539)	5,712 (1,662)	<.0001
Total (extrapolated) 1‐year cost (€) using the actual average number of 8 cures per patient per year in the hospital group and 8.4 in the home group	91,798 (51,125) [74,750]	48,189 (26,105) [44,148]	[24,723; 59,849] <.0001
Patients with CIDP *N* = 8			
Total (extrapolated) 1‐year cost (€)	102,296 (36,968) [99,072]	47,823 (28,803) [37,661]	<.0001
Patients with MMN *N* = 9			
Total (extrapolated) 1‐year cost (€)	70,747(24,098) [63,571]	37,338 (12,939) [44,902]	<.0001
Patients with Lewis–Sumner syndrome *N* = 7			
Total (extrapolated) 1‐year cost (€)	106,867 (81,082) [76,020]	62,592 (31,677) [55,741]	<.0001

GP, general practitioner; CIDP, chronic inflammatory demyelinating polyradiculoneuropathy; MMN, multifocal motor neuropathy.

## 4 DISCUSSION

Home‐based IVIg for treatment of primary (PID) or secondary immunodeficiency (SID) at a low dose (0.4 g/kg/cycle) has been used since the 1990s (Ochs et al., 1987; Ryan et al., 1988) in Europe and North America. As early as 1994, use of high doses of IVIg at home was authorized in the Netherlands for the treatment of immune neuropathies (Cats, Van der Pol, Bertens, & van den Berg, 2011), subject to certain criteria being me prior to treatment. These included administration of at least one cycle of IVIg (corresponding to a cumulative dose of 2 g per kg per cycle) in hospital, the presence of a nurse specialized in home management during the last cycle administered in hospital, prescription of an anti‐allergic reaction kit (epinephrine, prednisone, and an antihistamine), blood pressure monitoring, and verification of the possibility of a venous access port. During the same period, home infusion was not yet a widespread practice in patients with autoimmune diseases in France and many other European countries, probably due to a lack of experience among hospital practitioners and/or concern about adverse events (AEs). Even though IVIg is generally safe, serious adverse events such as thromboembolic events (Marie, Maurey, Hervé, Hellot, & Levesque, 2006; Rajabally & Kearney, 2011) or renal failure (Caress, Kennedy, & Eickman, 2010) can still occur, especially in patients treated by a high dose of IVIg or with concomitant diseases. In this analysis, the mean number of AEs was not statistically different between hospital and home treatment, and most AEs were ranked as mild or moderate. Home administration of IVIg began in France in the 1990s as a cost‐saving measure, as well as for the benefit of patients’ comfort and quality of life (Hachulla et al., 2002)*,* and it is now considered to be a safe alternative to hospital‐based treatments.

Home‐based IVIg as maintenance therapy clearly reduced hospital costs in our group of patients with autoimmune neuropathies. Total yearly treatment costs were divided roughly in half, from an average of €91,000 down to €48,000 per patient, with consistent savings across all three indications. Cost savings were achieved through fewer admissions and, to a much lesser extent, fewer commutes. Lower cost of MMN patients was explained by more outpatient hospital care.

Patients were satisfied by the switch to home treatment and experienced a small but significant reduction in their mRankin score. However, only patients that had already tolerated home‐based treatment and wanted to continue were included, which might bias the results toward a positive opinion on home treatment. We did not attempt to establish a causal relationship between satisfaction and home switch as with a randomized trial, but rather to propose a cost‐reducing alternative for selected patients.

The economic literature on immune neuropathies is scarce, and our findings are consistent with those of other authors who found an average yearly cost of £49,430 per patient on immunoglobulin, with an average IVIg dose per infusion close to our own. Of note, patients in the UK more frequently used outpatient hospital services than did patients in France, which explained a slightly lower cost (Mahdi‐Rogers, McCrone, & Hughes, 2014).

In an Italian study which provided a detailed calculation of the actual costs for in‐hospital IVIg treatment, the yearly cost was estimated to be €50,895 per year. The higher hospital cost in our analysis was explained by (1) the actual length of stay of 2.8 days (compared to 2 days in the Italian study) and (2) the inclusion of hospital overheads in our cost calculations. Lazzaro et al. estimated the costs of IVIg administration from a societal perspective and reported the itemized costs to the healthcare system s, that is, professional time and drugs and tests. We used total hospital costs which included all the logistics, maintenance, housekeeping, and support functions such as general administration, pharmacy, and sterilization. Including these costs resulted in a 30%–50% increase over direct hospital costs (Lazzaro, Lopiano, & Cocito, 2014).

Another route to reduce cost is optimization of IVIg dose based on patient response, which could also be combined with home‐based care, or switch to subcutaneous treatment which, when feasible, also results in major cost savings (Cocito et al., 2012; Lunn et al., 2016). We did not consider treatment with steroids, which would be always cheaper than IVIg, because the purpose of this analysis was to examine the most efficient delivery of IVIg in patients who cannot be treated with steroids, either because of previous treatment failure or because of contraindication.

Although our cost minimization analysis is innovative in that very limited cost data are available on immune neuropathies, it does have limitations. Our cost minimization analysis was undertaken on a small population, which is explained by both the rarity of the disease and the selection of stable patients who could be switched to home treatment. It was designed as a before–after study with no randomization and would be useful to policymakers only insofar as “switchable” population could be identified and be large enough to justify investing in home‐based treatment.

The entry costs of setting up a program for home‐based treatment have not been collected, but include time costs involved in providing information to patients and professionals, remote monitoring of patients’ treatment via telephone call or emails, and other hidden costs related to changing practice.

The estimated proportion of patients who could benefit from the switch was estimated to be 20% of all CIDP patients depending on both medical (disease stability) and demographic (age and ability to use monitoring tools) characteristics, as well as the environment (distance from the hospital and availability of nurses). Among patients with stable disease, the proportion could be as high as 80%.

Despite its limitations, this analysis has shown that the switch from hospital‐based to home‐based treatment for patients with immune neuropathy potentially represents significant savings in the management of the disease.

## CONFLICT OF INTEREST

Dr. Delmont has nothing to disclose. Dr. Sole reports grants from LFB Biomédicaments, during the conduct of the study; grants and non‐financial support from LFB Biomedicaments, grants, personal fees and non‐financial support from CSL‐Behring, grants and non‐financial support from Sanofi Genzyme, grants from Pfizer, outside the submitted work. Dr. Desnuelle has nothing to disclose. Dr. Durand‐Zaleski reports personal fees from LFB, during the conduct of the study; and president of the scientific committee of the French blood transfusion organization. Dr. Le Masson reports personal fees from LFB Biomedicament, during the conduct of the study; personal fees from LFB Biomedicament, outside the submitted work. Dr Puget has been working at LFB. Dr. Gauthier‐Darnis is an employee of LFB.

## AUTHORS’ CONTRIBUTIONS

All authors contributed to the design of the study; GS and GLM collected the data; IDZ, GLM, MGD, and SP analyzed the research; IDZ and GLM drafted the manuscript; and all authors reviewed and accepted the content of the manuscript prior to its submission.
